# Effects of rehabilitation training of core muscle stability on stroke patients with hemiplegia

**DOI:** 10.12669/pjms.36.3.1466

**Published:** 2020

**Authors:** Xiaofeng Chen, Zhuohui Gan, Wuchao Tian, Yongkai Lv

**Affiliations:** 1 Xiaofeng Chen, Department of Rehabilitation Medicine, Shenzhen Baoxing Hospital, Shenzhen 518115, P. R. China; 2 Zhuohui Gan, Department of Internal Medicine, The First Military Honor Rehabilitation Hospital of Guangdong, Guangzhou 510260, P. R. China; 3 Wuchao Tian, Department of Rehabilitation Medicine, Shenzhen Baoxing Hospital, Shenzhen 518115, P. R. China; 4 Yongkai Lv, Department of Rehabilitation Medicine, Shenzhen Baoxing Hospital, Shenzhen 518115, P. R. China

**Keywords:** Ambulation ability, Abdominal muscle thickness, Balance function, Core muscle stability, Rehabilitation training, Stroke

## Abstract

**Objective:**

To evaluate the effects of rehabilitation training of core muscle stability on stroke patients with hemiplegia.

**Methods:**

A total of 180 stroke patients who were hospitalized from December 2017 to December 2018 were enrolled. They were randomly divided into an observation group and a control group (n=90) that both received conventional hemiplegia rehabilitation therapy. On this basis, the observation group was subjected to training for core muscle stability, five times a week for a total of eight weeks. The balance functions before and after training were assessed using the Berg Balance Scale (BBS). The functions of hemiplegic lower limbs were evaluated by Brunnstrom staging and the Fugl-Meyer motor assessment (FMA) scale. The walking speed was estimated using the 10 m walking test. Musculoskeletal ultrasonography was performed to measure the thicknesses of three abdominal muscles of the paralytic side, i.e. transverse abdominis, internal oblique and external oblique muscles.

**Results:**

After treatment, the BBS scores of the two groups were significantly higher than those before treatment, with significant differences (P<0.05). The BBS score of the observation group was significantly higher than that of the control group (P<0.05). After treatment, the Brunnstrom stage and FMA scale score, and standing and stepping scores were significantly higher than those before treatment (P<0.05). The Brunnstrom stage, FMA scale score, stepping score and walking speed of the observation group significantly exceeded those of the control group (P<0.05). After treatment, the thicknesses all increased compared with those before treatment, but the thicknesses of internal oblique and external oblique muscles were not significantly different (P>0.05). The thickness of transverse abdominis muscle of the observation group significantly surpassed that before treatment (P<0.05), whereas the thicknesses of the control group were similar (P>0.05). The thickness of transverse abdominis muscle of the observation group was significantly higher than that of the control group (P<0.05).

**Conclusion:**

Rehabilitation training of core muscle stability can effectively improve the balance function and walking speed of stroke patients, probably by increasing the thickness of transverse abdominis muscle.

## INTRODUCTION

Stroke survivors often have multiple dysfunctions which seriously affect their daily life, work and social communications. The decline in balance function is one of the common dysfunctions.^1^ Training the stability of core muscles can improve the balance function of stroke patients, which is maintained by various factors.^2^,^3^ The increase of core muscle strength is one of the most important factors, which, however, cannot be easily measured due to subjectivity, interference factors and low credibility of the results.^4^ For a specific muscle, the strength is directly proportional to its cross-sectional area. Therefore, musculoskeletal ultrasonography has been used to measure the thickness of muscle, which is objective, non-invasive and low-cost.^5^ Until now, the trunk muscles of stroke patients have seldom been tested by ultrasonography. Besides, previous studies have verified that core muscle training can improve the balance ability of stroke patients, but the part of human body responsible for this improvement remains largely unknown.^6^,^7^ Thereby motivated, we herein evaluated the effects of rehabilitation training of core muscle stability on the balance function, ambulation ability and abdominal muscle thickness of stroke patients with hemiplegia, aiming to provide valuable clinical evidence for their treatment.

## METHODS

### Baseline Clinical Data

This study has been approved by the ethics committee of our hospital, (Dated on December 4, 2017) and written informed consent has been obtained from all cases. A total of 180 stroke patients who were hospitalized from December 2017 to December 2018 were enrolled.

### Inclusion criteria

1) In accordance with the diagnostic criteria for stroke formulated at the 4th National Conference on the Diagnosis of Cerebrovascular Diseases,^8^ with confirmation by head CT or MRI; 2) first onset; 3) course within 6 months with stable conditions; 4) with ability to understand the instructions of researchers, and score of mini-mental state examination scale of ≥24 points; 5) with ability to maintain a standing position for over one minute in the case of eye opening.

### Exclusion criteria

1) With serious heart, lung, liver, kidney and other diseases of vital organs, as well as unstable vital signs; 2) with other nervous system diseases causing balance dysfunction; 3) with severe orthopedic diseases affecting standing; 4) with serious cognitive, speech or vision disorder to be unable to complete this study; 5) underweight (BMI <18.5) or overweight (BMI ≥24). The 180 included patients were randomly divided into an observation group and a control group (n=90). There were no significant differences in gender, age, BMI, duration of disease, as well as nature and site of lesions between the two groups (P>0.05) (Table-I).

**Table-I:** Baseline clinical data.

	Observation group (n=90)	Control group (n=90)	t/χ^2^	P
Age (year)	59.12±12.67	59.05±12.74	0.037^a^	0.971
BMI (kg/m^2^)	24.37±2.56	23.96±2.61	1.064^a^	0.289
Gender			0.095^b^	0.758
Male	57	55		
Female	33	35		
Disease course (d)	23.79±2.45	24.06±2.53	0.727^a^	0.468
Lesion nature			0.092^b^	0.762
Cerebral infarction	54	52		
Cerebral hemorrhage	36	38		
Lesion site			0.091^b^	0.763
Left side	39	37		
Right side	51	53		

a: t value; b: χ^2^ value.

### Treatment Methods

The observation group was given routine rehabilitation training combined with core muscle training, and the control group was given conventional rehabilitation training combined with trunk control training.

### (1) Routine rehabilitation training

1) Position of the non-affected limb; 2) physical therapy based on Bobath technology; 3) sitting position and standing balance training; 4) gait decomposition training; 5) daily life activity training. All trainings were performed once a day, 40 min each time, 6 days per week for 8 consecutive weeks.

### (2) Core muscle training

Core muscle training was performed using a multi-point multi-axis suspension training system. 1) The patient was placed in the supine position, the knee joints of the lower limbs were suspended with inelastic suspension straps, and the pelvis was raised to the horizontal position and maintained; 2) the patient was placed in the supine position, the waist was assisted by elastic suspension straps, and the lower extremity ankle joints were suspended with inelastic suspension straps, and the pelvis was raised to a horizontal position and maintained; 3) the patient was placed in the supine position, the waist was assisted by elastic suspension straps, and the affected lower extremity ankle joint were suspended with the inelastic suspension straps, and the pelvis was raised to a horizontal position and maintained; 4) the patient was placed in the lying position of the affected side, the waist was assisted by elastic suspension straps, the affected knee joint was suspended with inelastic suspension straps, the pelvis was raised to the horizontal position and maintained; 5) the patient was placed in a prone position, with the support of double elbows, the waist was assisted by elastic suspension straps, the knees and ankle joints were suspended with inelastic suspension straps, and the pelvis was raised to the horizontal position and maintained. All trainings were performed once a day, 40 minutes each time, 6 days per week for 8 consecutive weeks. During the training, the patient maintained normal breathing, and the duration of each movement was gradually increased from the tolerance (5~10 s). The therapist gradually extended the maintenance time according to the patient’s specific condition, with the maximum length not exceeding three minutes.

### (3) Truck control training

1) Roll-up training under supine position, bridge movement, sit-up training; 2) sitting-position trunk flexion and extension and rotation training; 3) resistance training of sitting-position trunk flexion and extension and rotation. All trainings were performed once a day, 40 min each time, six days per week for 8 consecutive weeks.

### Evaluation Indices

(1) Assessment of balance ability: The Berg Balance Scale (BBS) was used to evaluate the balance function before and after test. There were 14 items, each with a maximum of 4 points and a minimum score of 0. A higher score meant better balance function.
(2) Evaluation of lower limb function and walking speed: The functions of hemiplegic lower limbs were evaluated by Brunnstrom staging and the Fugl-Meyer motor assessment (FMA) scale. The walking speed was estimated using the 10 m walking test.
(3) Ultrasonography of abdominal muscles: Musculoskeletal ultrasonography was performed to measure the thicknesses of three abdominal muscles of the paralytic side, i.e. transverse abdominis, internal oblique and external oblique muscles. A high-frequency linear array probe (5~12 MHz) and Voluson S8 color Doppler ultrasound system (General Electric, USA) were employed. The patient was in the supine position and fully relaxed. The ultrasound probe was placed at the midpoint of the 12th rib and the anterior superior iliac spine. The direction of the probe was slightly adjusted. The three muscles to be observed could be clearly seen, with the joint between the transverse abdominis muscle and the outermost part of the thoracolumbar fascia as the anchor point, and the muscle fibers along the transverse abdominis muscle two cm to the inside as the measurement points for the three muscles. The vertical distance between the two layers of linear echogenic fascia was the thickness of the muscle measured.^9^ To attenuate the influence of breathing on muscle thickness, measurements were carried out at the end of calm exhalation. Each muscle was measured 3 times and the average was recorded.

Balance ability, lower limb function and walking speed were evaluated by the same rehabilitation therapist, and ultrasonography was performed by the same sonographer. All tests were conducted in a single-blinded manner, i.e. the operators were unaware of study grouping or treatment methods.

### Statistical Analysis

All data were analyzed by SPSS 16.0 software. The continuous data were expressed as mean ± standard deviation. Comparisons within the same group before and after treatment were performed by the paired t test, and intergroup comparisons were conducted with the independent sample t test. The categorical data were compared by using the χ^2^ test. P<0.05 was considered statistically significant.

## RESULTS

Both groups successfully completed this study. Before treatment, the BBS scores of the two groups were similar (P>0.05). After treatment, the BBS scores of the two groups were significantly higher than those before treatment, with significant differences (P<0.05). The BBS score of the observation group was significantly higher than that of the control group (P<0.05) (Table-II).

**Table-II:** BBS scores before and after treatment (point).

	Observation group (n=90)	Control group (n=90)
Before treatment	21.74±2.02	22.03±1.97
After treatment	37.98±3.42*,#	32.68±3.15*

* : Comparison within the same group before and after treatment, P<0.05;

#: comparison between observation and control groups after treatment, P<0.05.

Before treatment, there were no significant differences in the Brunnstrom stage, FMA scale score, and sitting, standing and stepping scores between the two groups (P>0.05). After treatment, the Brunnstrom stage and FMA scale score, and standing and stepping scores were significantly higher than those before treatment (P<0.05). The Brunnstrom stage, FMA scale score, stepping score and walking speed of the observation group significantly exceeded those of the control group (P<0.05) (Table-III).

**Table-III:** Lower limb function and walking speed.

	Brunnstrom stage of hemiplegic lower limb	FMA scale score (point)	Total score of Brunel balance assessment scale (point)	Sitting (0-3)	Standing (0-3)	Stepping (0-6)	10m walking test (m/s)
*Observation group (n=90)*							
Before treatment	2.37±0.23	11.69±1.24	4.43±0.32	3.00±0.00	1.41±0.15	0.00±0.00	NT
After treatment	4.45±0.25*,#	23.97±2.12*,#	10.18±0.48*,#	3.00±0.00	3.00±0.00*	4.17±0.32*,#	0.51±0.09#
*Control group (n=90)*							
Before treatment	2.41±0.22	11.71±1.31	4.44±0.29	3.00±0.00	1.42±0.16	0.00±0.00	NT
After treatment	4.02±0.26*	21.25±2.34*	8.76±0.52*	3.00±0.00	3.00±0.00*	2.84±0.53*	0.42±0.07

* : Comparison within the same group before and after treatment, P<0.05;

#: comparison between observation and control groups after treatment, P<0.05.

There were no significant differences in the thicknesses of transverse abdominis, internal oblique and external oblique muscles between the two groups before treatment (P>0.05). After treatment, the thicknesses all increased compared with those before treatment, but the thicknesses of internal oblique and external oblique muscles were not significantly different (P>0.05). The thickness of transverse abdominis muscle of the observation group significantly surpassed that before treatment (P<0.05), whereas the thicknesses of the control group were similar (P>0.05). The thickness of transverse abdominis muscle of the observation group was significantly higher than that of the control group (P<0.05) (Table-IV).

**Table-IV:** Thicknesses of transverse abdominis, internal oblique and external oblique muscles.

	Thickness of transverse abdominis muscle (mm)	Thickness of internal oblique muscle (mm)	Thickness of external oblique muscle (mm)
*Observation group (n=90)*			
Before treatment	2.22±0.21	5.45±0.78	3.41±0.31
After treatment	2.79±0.24*,#	5.51±0.81	3.47±0.32
*Control group (n=90)*			
Before treatment	2.23±0.23	5.43±0.75	3.42±0.33
After treatment	2.25±0.21	5.48±0.71	3.46±0.32

* : Comparison within the same group before and after treatment, P<0.05;

#: comparison between observation and control groups after treatment, P<0.05.

## DISCUSSION

Stroke is a neurological disease. There are different degrees of dysfunction after stroke, which has seriously threatened people’s health. Early improvement of motor function and balance in stroke patients is important for disease recovery. Currently, patients with hemiplegia due to stroke at home and abroad mainly use rehabilitation function training, and the methods are more and the curative effect is different.

Balance ability is the ability to coordinate the stimuli from the vestibular organs, muscles, tendons, joints, and vision. It is the basic premise for people to maintain posture and accurately complete technical movements. Disorders in the motor or sensory pathways of patients after stroke lead to abnormal muscle tone and muscle strength and motor control disorders, eventually resulting in balance dysfunction. Whether the balance function restores the walking ability and daily living ability of patients with hemiplegia due to cerebral infarction.^10^ Core stability refers to controlling the steady state of the muscles in the pelvis and trunk during exercise, creating a fulcrum for the movement of the upper and lower limbs, and coordinating the force of the upper and lower limbs to optimize the generation, transmission and control of power. Core stability training can improve the body’s ability to control and balance, articulate, transmit and integrate the power output of each muscle group. Therefore, researchers have endeavored to develop methods to enhance the core strength and stability.^11^

Recently, core stability training has received widespread attention in the field of rehabilitation medicine. Core stability training can improve the daily living ability and walking ability of stroke patients, and help to promote their return to family and society.^12^

At present, there are limited methods for evaluating the muscle strength of trunk core muscles. Although the electromyogram can be used for the examination of abdominal muscles, because the intra-abdominal oblique muscles and the transverse abdominis muscles have overlapping muscle fibers. In addition, there is a certain error in the placement of the body surface electrodes, so the accuracy of myoelectric signal is low and the clinical application is limited. Musculoskeletal ultrasonography can avoid these shortcomings.^9^ Musculoskeletal ultrasonography not only has no ray damage, low price, non-invasive, but also has the advantages of real-time imaging, short-term repeatable examination and high soft tissue resolution.^13^ For rehabilitation training, musculoskeletal ultrasound cannot only shorten the clinical diagnosis time, but also provide more direct evidence for the judgment of clinical rehabilitation. Although musculoskeletal ultrasonography is currently the focus of research, the use of musculoskeletal ultrasound imaging to assess the morphological changes in the core muscles of stroke patients and its relationship with balance is relatively rare.

The core muscle training techniques used in this study are mostly performed in unstable environments (balance pads, training balls), and the flexion, extension and rotation training of the trunk are performed in different positions (station, lying position, sitting position). It involves deep and superficial waist and abdomen core muscles, and the training is more comprehensive and more targeted. After treatment, the BBS scores of the two groups were significantly higher than those before treatment, with significant differences (P<0.05). The BBS score of the observation group was significantly higher than that of the control group (P<0.05). Therefore, routine rehabilitation training can improve the balance function of stroke patients, but training for core muscle stability improved the balance ability more effectively, being consistent with a previous literature.^14^ After treatment, the Brunnstrom stage and FMA scale score, and standing and stepping scores were significantly higher than those before treatment (P<0.05). The Brunnstrom stage, FMA scale score, stepping score and walking speed of the observation group significantly exceeded those of the control group (P<0.05). Thus, core muscle training further enhanced the control ability and balance function of the trunk, then elevating the walking speed.

The trunk core muscles comprise local and overall stabilizing muscles. In this study, transverse abdominis, internal oblique and external oblique muscles were selected as the targets for ultrasonography, because they play dominant roles in the posture control of the trunk. The trunk muscles are controlled by bilateral pyramidal tracts. Bae et al. reported no significant differences between the thicknesses of bilateral abdominal muscles in stroke patients.^15^ After treatment herein, the thicknesses all increased compared with those before treatment, but the thicknesses of internal oblique and external oblique muscles were not significantly different (P>0.05). The thickness of transverse abdominis muscle of the observation group significantly surpassed that before treatment (P<0.05), whereas the thicknesses of the control group were similar (P>0.05). The thickness of transverse abdominis muscle of the observation group was significantly higher than that of the control group (P<0.05). The results further verified the importance of the transverse abdominis muscle as a local stabilizing muscle, suggesting that this muscle should be specifically trained to augment the balance ability. The thicknesses of internal oblique and external oblique muscles hardly changed after training, which may be related to their limitations in maintaining trunk stability^16^,^17^ or the design, training intensity and small sample size of this study.

In summary, rehabilitation training of core muscle stability can effectively improve the balance function and walking speed of stroke patients, probably by increasing the thickness of transverse abdominis muscle. However, this study has some limitations, such as small sample size and low number of involved core muscles. Further in-depth studies on ongoing in our group.

### Authors’ Contributions:

**XC & ZG:** Study design and significant manuscript revision. The two authors contributed equally to this study.

**WT & YL:** Manuscript drafting, clinical data collection and analysis.

**XC, ZG, WT & YL:** Approval of manuscript submission.

**All authors:** Responsible and accountable for the accuracy or integrity of the work.
